# Stable and efficient generation of functional iPSC-derived neural progenitor cell rosettes through regulation of collective cell-cell behavior

**DOI:** 10.3389/fbioe.2023.1269108

**Published:** 2024-01-10

**Authors:** Mee-Hae Kim, Naruchit Thanuthanakhun, Masahiro Kino-oka

**Affiliations:** ^1^ Department of Biotechnology, Graduate School of Engineering, Osaka University, Osaka, Japan; ^2^ Research Base for Cell Manufacturability, Osaka University, Osaka, Japan

**Keywords:** human induced pluripotent stem cells, neural progenitor differentiation, neural rosette, botulinum hemagglutinin, cell behavior

## Abstract

Although the potential of stem cells to differentiate into several cell types has shown promise in regenerative medicine, low differentiation efficiency and poor reproducibility significantly limit their practical application. We developed an effective and robust differentiation strategy for the efficient and robust generation of neural progenitor cell rosettes from induced pluripotent stem cells (iPSCs) incorporating botulinum hemagglutinin (HA). Treatment with HA suppressed the spontaneous differentiation of iPSCs cultured under undirected differentiation conditions, resulting in the preservation of their pluripotency. Moreover, treatment with HA during neural progenitor differentiation combined with dual SMAD inhibition generated a highly homogeneous population of PAX6-and SOX1-expressing neural progenitor cells with 8.4-fold higher yields of neural progenitor cells than untreated control cultures. These neural progenitor cells formed radially organized rosettes surrounding the central lumen. This differentiation method enhanced the generation of functional iPSC-derived neural progenitor cell rosettes throughout the culture vessel, suggesting that the regulation of collective cell-cell behavior using HA plays a morphogenetically important role in rosette formation and maturation. These findings show the significance of HA in the suppression of spontaneous differentiation through spatial homogeneity. The study proposes a novel methodology for the efficient derivation of functional iPSC-derived neural progenitor cell rosettes.

## Introduction

The application of human pluripotent stem cells (PSCs) as renewable sources of specialized cells is limited by the lack of robust and highly efficient differentiation strategies (Robinton and Daley, 2012; Chen et al., 2014; Ohnuki and Takahashi, 2015). Most of the proposed culture strategies for generating the embryonic germ layers from embryonic stem cells (ESCs) and induced pluripotent stem cells (iPSCs) have relatively low differentiation efficiency and poor reproducibility (Ohnuki and Takahashi, 2015; Kim and Kino-oka, 2018; Kim and Kino-oka, 2020). Until recently, these strategies involved stepwise addition of soluble molecules, including growth factors and small molecules, at specific time points during certain steps of differentiation (Chambers et al., 2009; Madhu et al., 2016; Lukovic et al., 2017; Zhang et al., 2018). However, in most cases, the spatiotemporal patterns of population changes within and across cultures result in heterogeneous stem cell fates during directed differentiation even under homogeneous bulk culture conditions (Kim et al., 2021; Thanuthanakhun et al., 2022; Kim et al., 2023). This necessitates cell behavior control to avoid generating spatial heterogeneity, which may trigger differentiation, and to spatially control cell behavior, consequently improving the generation of functionally differentiated cells during directed differentiation.

Understanding the effect of different environmental cues on decision rules during cell-fate instruction facilitates more optimized control of PSC proliferation and differentiation by developing culture strategies using biomaterials as culture tools. Spatial patterns of cell growth and differentiation are often associated with intracellular phenomena, particularly cellular behavioral changes (cell-cell interactions, cell-substrate interactions, and cell migration) (Weber et al., 2011; Kim and Kino-oka, 2014; Kim and Kino-oka, 2015; Shuzui et al., 2019). Several studies on ESCs and iPSCs have shown that integrin and E-cadherin adhesion can be transmitted throughout the interconnected cytoskeleton through cytoskeletal anchor forces (Kim and Wirtz, 2015). Integrin- and cadherin-mediated adhesions spatiotemporally coordinate the formation of tension-high adherens junctions, providing mechanical connections between the nucleus and the cytoskeleton (Dupont, 2016; Mui et al., 2016; Dasgupta and McCollum, 2019). Such processes can trigger distinct downstream signaling cascades that regulate gene expression and direct cell fate decisions (Dupont, 2016; Dasgupta and McCollum, 2019). Normal and conventional cell cultures exhibit variations in the size, shape, and density of colonies during PSC culture, suggesting inhomogeneity in the mechanical forces and topological cell relationships within and between cultures (Deglincerti et al., 2016; Heemskerk et al., 2019; Petzold and Gentleman, 2021; Kim et al., 2022). Several models explain how these differences emerge from different topological cell features, including differential expression patterns of genes involved in morphogen signaling and differences in the cytoskeletal contractile force (Deglincerti et al., 2016; Kim et al., 2022). In micropatterned PSC colonies, cells in the colony periphery exhibit relatively high cell-substrate interactions with well-established integrin-based focal adhesions, whereas cells in the colony center exhibit relatively high E-cadherin-mediated cell-cell interactions (Deglincerti et al., 2016; Kim et al., 2022). These topological differences in PSC colonies influence cell fate specification and patterning (Deglincerti et al., 2016; Heemskerk et al., 2019). However, despite the biological importance of spatial mechanical variations, the precise mechanisms by which cells within a culture affect cellular fate determination are unclear. The lack of adequate culture systems for the control of spatial heterogeneity, a major impediment to the development of a robust and highly efficient culture strategy, contributes to this lack of clarity.

Controlling iPSC behavior using botulinum hemagglutinin (HA) has been identified as a culture strategy for controlling iPSC self-renewal and differentiation potential (Kim et al., 2017; Shuzui et al., 2019; Kim et al., 2023). HA is composed of three subcomponents: HA1, HA2, and HA3. HA has at least two biological activities: carbohydrate and E-cadherin binding, and directly binds to the EC1-EC2 domain of E-cadherin via transcellular and paracellular routes, inhibiting its dimerization and disrupting the epithelial barrier (Lee et al., 2014; Sugawara et al., 2014; Amatsu et al., 2018). The loss of E-cadherin-mediated adhesion facilitates the spatial dispersion of cells, resulting in the suppression of spatial heterogeneity (Shuzui et al., 2019; Kim et al., 2023). When iPSCs form compact colonies, the disruption of E-cadherin binding between cells by treatment with HA leads to mechanical memory synchronization with YAP proteins (Kim et al., 2023). This effect of HA-mediated E-cadherin adhesion disruption is transient and can be controlled by varying the HA concentration and exposure time. Therefore, the use of HA during directed differentiation may facilitate the production of a more uniform population of differentiated cells by directly controlling spatial heterogeneity within the culture.

In this study, we established an efficient and robust strategy for the direct differentiation of iPSCs into neural progenitors. We demonstrated that treatment of HA suppresses spatial heterogeneity in cell differentiation induction, and dual inhibition of TGF-β/Activin/Nodal and BMP signaling can improve functional neural progenitor differentiation. The spatiotemporal control of cell behavior using HA can yield a large number of rosette-forming neural progenitors. Therefore, the differentiation strategy combined with HA, involving the suppression of unwanted spontaneous differentiation, is a relatively efficient and convenient method for enriching neural progenitor cultures. Moreover, the incorporation of this strategy into previously existing neuron differentiation strategies will considerably improve the quality and consistency of the resulting neuronal populations. This insight allows for the tailoring of biomaterials for the highly efficient generation of functional neural rosettes from PSCs throughout the culture vessel.

## Materials and methods

### Maintenance and differentiation of iPSCs

The human iPSC line 1383D6 (Center for iPS Cell Research and Application, Kyoto University, Kyoto, Japan) was maintained on laminin 511-E8-coated dishes (iMatrix-511; Nippi, Inc., Japan) in a chemically defined medium (StemFit AK02N; Ajinomoto Co., Inc., Japan), as described in a previous study (Nakagawa et al., 2014). For subculturing, single cells were seeded with 10 μM Rho-associated coil containing the kinase (ROCK) inhibitor Y-27632 (Fujifilm Wako Pure Chemical, Japan). The cells were treated with 5 mM ethylenediaminetetraacetic acid/phosphate-buffered saline (PBS) with 10 μM Y-27632 for 7 min at room temperature, then dissociated into single cells using TrypLE Select (Invitrogen, USA) in 10 μM Y-27632. They were then seeded at a viable cell density of 7.5 × 10^3^ cells/cm^2^ and incubated at 37°C in a humidified atmosphere with 5% CO_2_. The medium was replaced daily with a fresh one.

**FIGURE 1:**
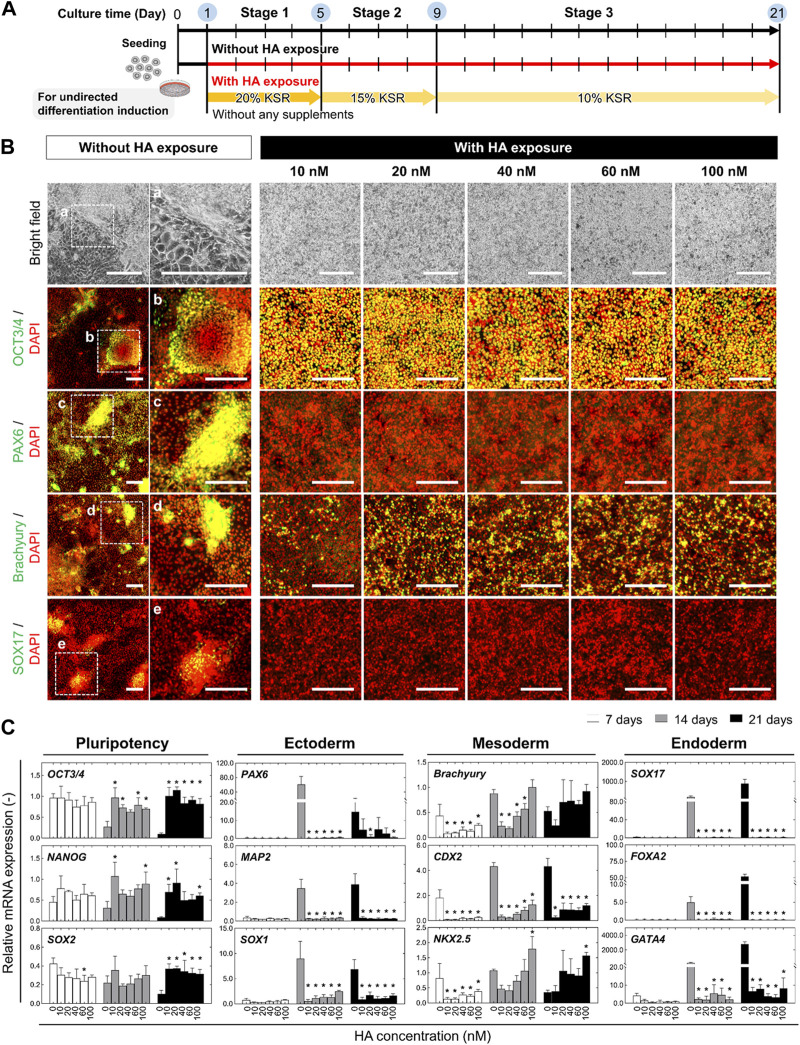
Morphological and phenotypic characterization of undirected differentiation of iPSCs with or without HA. **(A)** Experimental scheme to examine the impact of exposure to HA during undirected differentiation of iPSCs. Cells were cultured on iMatrix-coated surface in KSR-based medium free of any additional soluble signaling factors for 21 days. The different concentrations of HA (10, 20, 40, 60, and 100 nM) were repeatedly exposed to cells from days 1–14. **(B)** Representative bright-field images showing morphological changes and immunofluorescent images of pluripotency and trilineage differentiation markers including OCT3/4, PAX6, Brachyury, and SOX17 on day 21 of undirected differentiation. Nuclei were stained with DAPI. Panels a–e are the enlarged images of the boxed area in the merged panels. Scale bars, 500 μm. **(C)** Relative mRNA expression of pluripotency-associated genes (*OCT3/4, NANOG,* and *SOX2*) and early lineage specification-associated genes of ectoderm (*PAX6, MAP2* and *SOX1*), mesoderm (*Brachyury, CDX2* and *NKX2.5*), and endoderm (*SOX7, FOXA2,* and *GATA4*) at different time points (on days 7, 14, and 21) of undirected differentiation. Fold-expression for each mRNA obtained for each method was normalized to that of undifferentiated iPSCs on day 0. Data are presented as mean ± SD (*n* = 3). Significance was determined using one-way ANOVA with Tukey’s test (*p-value <0.01, compared to control).

The procedure used for the undirected and directed differentiation of iPSCs is shown in Figures 1A, 3A. Briefly, undifferentiated hiPSCs were seeded at a density of 1.0 × 10^5^ cells/cm^2^ onto 6-well plates coated with iMatrix in StemFit AK02N medium supplemented with 10 μM Y-27632 (day 0). During differentiation, the basal medium contained Glasgow Minimum Essential Medium (Sigma-Aldrich, USA) and was supplemented with knockout serum replacement (KSR; Invitrogen) at a particular concentration, 1 mM sodium pyruvate, 1 mM L-glutamine, 1% non-essential amino acids (NEAA), 0.1 mM 2-mercaptoethanol, 50 μg/mL penicillin, and 50 μg/mL streptomycin. From days 1–20, the medium was changed to the differentiation medium according to the following criteria.

For undirected and directed differentiation of iPSCs, the cells were cultured for 4 days in 20% KSR-based medium, then in 15% KSR-based medium for 4 days, and finally in 10% KSR-based differentiation medium for 12 days. Treatment of iPSCs with HA (0, 10, 20, 40, 60, and 100 nM) began on day 1 and continued throughout the culture period. The medium was changed every day.

For directed differentiation of iPSCs into neural progenitors, from days 1–9, the differentiation media were additionally supplemented with 500 nM LDN-193189 (a selective BMP signaling inhibitor; Sigma-Aldrich) and 10 μM SB431542 (a TGF-β receptor kinase inhibitor; Sigma-Aldrich). The treatment of iPSCs with 40 nM HA was initiated on day 1 and continued throughout the culture period. The medium was changed every day.

### Preparation of functional HA complex

Functional HA complexes were reconstituted and purified as described in previous reports (Sugawara et al., 2014; Amatsu et al., 2018). Briefly, each HA subcomponent was purified from *Escherichia coli*, and the functional HA complex was reconstituted by mixing the recombinant proteins (HA1, HA2, and HA3). All proteins were dialyzed in pH 7.4 PBS and stored at −80°C until further use. Protein concentrations were calculated using the BCA assay (Thermo Fisher Scientific, USA).

### Time-lapse observation

Neural differentiation of iPSCs was observed using a phase-contrast time-lapse observation incubator equipped with a camera (BioStudio T, Nikon, Japan). The imaging system was maintained at 37°C and 5% CO_2_ within the incubator. After manually selecting the viewing area, the system was set to automatically capture images every 30 min for 24 h using a ×4 objective lens.

### Immunofluorescence staining

Immunofluorescence staining was performed on fixed cells, as described in previous studies (Shuzui et al., 2019; Kim et al., 2023). Briefly, cells were washed with PBS and fixed with 4% paraformaldehyde (Fujifilm Wako Pure Chemical) for 10 min at room temperature. The cells were then washed with PBS and permeabilized with 0.5% Triton X-100 in PBS for 5 min, after which they were blocked for 90 min in Block Ace (Dainippon Sumitomo Pharma Co., Ltd., Japan). The cells were immersed at 4°C overnight with primary antibody and then incubated with secondary antibody for 1 h. Subsequently, they were washed three times with PBS and stained with 4’,6-diamidino-2-phenylindole (Thermo Fisher Scientific) for 10 min. Images were captured using an image analyzer with a ×10 objective lens (IN Cell Analyzer 2000; GE Healthcare, UK) or a confocal laser microscope (Model FV-1000; Olympus, Japan) with a ×60 objective lens. Primary and secondary antibodies used are listed in Supplementary Table S1.

### Western blot analysis

Western blot analysis was performed as previously described (Kim et al., 2023). The total cellular protein was extracted using RIPA lysis buffer (Sigma-Aldrich) supplemented with a protease and phosphatase inhibitor cocktail (Thermo Fisher Scientific). Total protein was quantified using the BCA assay. The proteins were dissolved in sodium dodecyl sulfate polyacrylamide gel electrophoresis buffer and transferred onto a polyvinylidene difluoride membrane. The membranes were blocked with 5% milk in Tris-buffered saline for 1 h at room temperature and then incubated with the following primary antibodies overnight at 4°C. After washing, the membranes were incubated with the secondary antibodies for 1 h at room temperature and rinsed to remove any residual detergent. β-actin was used as the internal loading control. Primary and secondary antibodies used are presented in Supplementary Table S1.

### Flow cytometry analysis

Flow cytometry analysis was performed as described in a previous study (Kim et al., 2023). Briefly, the cells were dissociated into single-cell suspensions using TrypLE Select. After fixation and permeabilization using a Cytofix/Cytoperm kit (BD Biosciences, USA) according to the manufacturer’s protocol, the cell suspension was stained with the fluorophore-conjugated antibodies for 30 min at 4°C in the dark. The cells were washed, resuspended in PBS, and analyzed using a flow cytometer (CyFlow Cube 6; Sysmex Partec, Germany). Primary and secondary antibodies used are presented in Supplementary Table S1.

### Quantitative real-time RT-PCR (qRT-PCR)

Total RNA was extracted using the RNeasy Mini Kit (Qiagen, Germany) according to the manufacturer’s instructions. For reverse transcription, the total RNA was used with a SuperScript II reverse transcriptase kit (Takara Bio, Japan), and real-time PCR was performed using SYBR Premix EX Taq (Takara Bio) on a 7300 real-time PCR system (Applied Biosystems, USA). β-actin was used as an endogenous control and mRNA expression, relative to that in untreated cells (control), was quantified by the 2^−ΔΔCT^ method. Primer sequences used are presented in Supplementary Table S2.

## Results

### Effect of HA on spontaneous differentiation of iPSCs in the absence of exogenous differentiation factors

Because HA-mediated temporal disruption of E-cadherin adhesion is a well-established method for controlling the spatial distribution of cells in iPSC culture (Shuzui et al., 2019; Kim et al., 2023), we investigated the effect of HA on the spontaneous differentiation of iPSCs in KSR-based medium without any exogenous differentiation factors. Various concentrations (0, 10, 20, 40, 60, and 100 nM) of HA were added during the medium change from days 1–21 (Figure 1A). The cells in the culture with HA exhibited a clear morphological distinction: they formed a population with a homogeneous morphology and multilayered structures after 21 days of differentiation, whereas the cells in the untreated (control) culture showed a mixture of monolayered areas and proliferative regions containing aggregates with irregular shapes (Figure 1B). In control cultures, several morphologically distinct cell populations emerged on day 21 of differentiation, including cells with different morphologies, such as squamous monolayer epithelial cells and fibroblast-like spreading cells. Immunofluorescence staining showed that the cells in the control culture showed a heterogeneous expression pattern for the pluripotency marker (OCT3/4), ectoderm marker (PAX6), ectoderm marker (PAX6), mesoderm marker (Brachyury), and endoderm marker (SOX17), leading to heterogeneity in differentiating iPSC cultures. However, most cells cultured in medium with HA expressed OCT3/4, indicating an undifferentiated state of iPSCs. These cells were slightly positive for brachyury, whereas most cells were negative for PAX6 and SOX17. The morphological changes and expression of the three lineages showed the same pattern regardless of the added concentration.

Consistent with the immunostaining results, RT-PCR analysis revealed similar expression profiles of lineage markers in differentiated iPSCs cultured with or without HA. Compared to undifferentiated iPSCs (day 0), the expression of pluripotency-associated genes (*OCT3/4, NANOG*, and *SOX2*) significantly decreased in the differentiation culture by day 21 (Figure 1C). Most early lineage specification-associated genes of the ectoderm (*PAX6, MAP2*, and *SOX1*), endoderm (*SOX7, FOXA2*, and *GATA4*), and mesoderm (*Brachyury, CDX2*, and *NKX2.5*) increased drastically by day 21. In contrast, their expression was markedly reduced in HA-treated cells. They maintained high expression of pluripotency-associated genes and suppressed the expression of lineage-specific genes compared to control cultures. The expression levels were not dependent on the HA concentration on days 7, 14, and 21. The cells in the control culture tended to spontaneously aggregate, leading to spontaneous differentiation of iPSCs. These results indicate that HA suppresses the spontaneous differentiation of iPSCs by controlling spatial heterogeneity.

**FIGURE 2:**
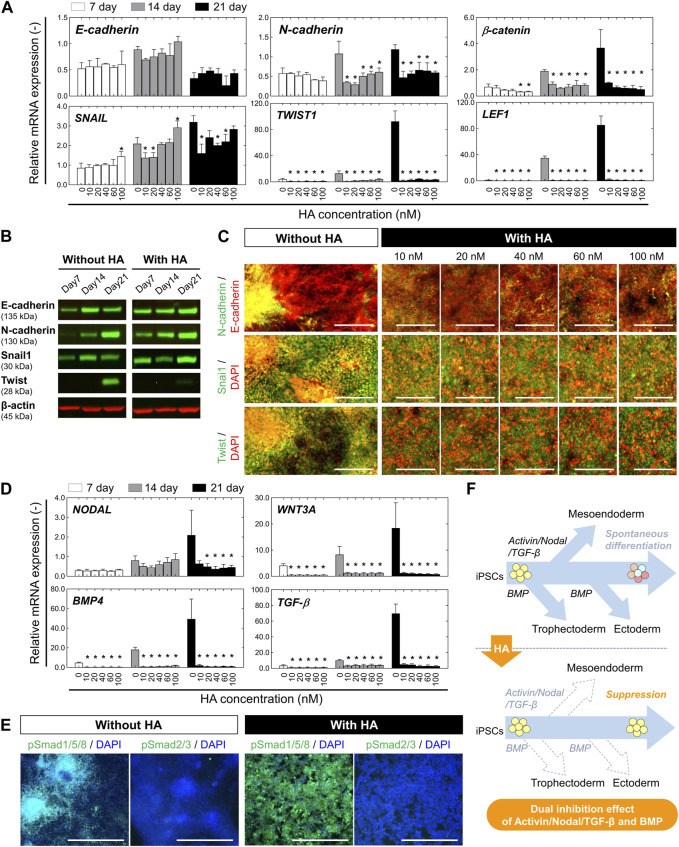
Characterization of EMT-associated signaling and TGF-β/Activin/Nodal and BMP-associated signaling during undirected differentiation of iPSCs with or without HA. **(A)** Relative mRNA expression of the epithelial marker (*E-cadherin*) and mesenchymal markers *(N-cadherin*, *β-CATENIN*, *SNAIL1*, *TWIST1*, and *LEF1*) at different time points (on days 7, 14, and 21) of undirected differentiation as shown in Figure 1A. Fold-expression for each mRNA obtained for each method was normalized to that of undifferentiated iPSCs on day 0. Data is presented as mean ± SD (*n* = 3). Significance was determined using one-way ANOVA with Tukey’s test (*p-value <0.01, compared to control). **(B)** Western blot analysis showing levels of E-cadherin, N-cadherin, Snail, and Twist at different time points (on days 7, 14, and 21) of undirected differentiation. β-actin was used as a loading control. The protein levels of E-cadherin, N-cadherin, Snail, and Twist were compared in culture conditions without or with HA (40 nM). **(C)** Immunofluorescent images of E-cadherin, N-cadherin, Snail, and Twist on day 21 of undirected differentiation. Nuclei were stained with DAPI. Scale bar, 200 μm. **(D)** Relative mRNA expression of TGF-β/Activin/Nodal and BMP-associated genes (*NODAL, WNT3A, BMP4,* and *TGF-β*) at different time points (on days 7, 14, and 21) of undirected differentiation. Fold-expression for each mRNA obtained for each method was normalized to that of undifferentiated iPSCs on day 0. Data is presented as mean ± SD (*n* = 10). Significance was determined using one-way ANOVA with Tukey’s test (*p-value <0.01, compared to control). **(E)** Immunofluorescent images of phospho-SMAD1/5/8 (pSMAD1/5/8) and phospho-SMAD2/3 (pSMAD1/5/8) on day 21 of undirected differentiation. Nuclei were stained with DAPI. The nuclear expressions of pSMAD1/5/8 and pSMAD2/3 were compared in culture conditions without or with HA (40 nM). Scale bar, 500 μm. **(F)** Schematic of the working hypothesis of repression of spontaneous differentiation from iPSCs through dual inhibition of TGF-β/Activin/Nodal and BMP signaling following HA-mediated E-cadherin disruption. Dashed arrows represent a decrease in expression for genes specific to the tissue indicated.

To assess whether the loss of E-cadherin expression after HA exposure was associated with epithelial-mesenchymal transition (EMT), we used qRT-PCR to measure the mRNA expression of major EMT-related transcription factors (*SNAIL*, *SLUG*, and *TWIST1*) and other regulatory molecules (*β-catenin* and *LEF1*) correlated with E-cadherin and N-cadherin expression on days 7, 14, and 21. Compared to the undifferentiated iPSC culture (day 0), the level of E-cadherin mRNA was significantly decreased on day 7, and to a greater extent in both culture conditions on day 21 (Figure 2A). We found that cells in the control culture showed a decrease in the expression of *E-cadherin* by day 21, which was consistent with the altered expression patterns of EMT genes (*SNAIL, SLUG, TWIST*, and *N-cadherin*). The mRNA levels of other pro-EMT genes (*β-catenin* and *LEF1*) significantly increased by day 21. In contrast, HA treatment resulted in the complete suppression of the expression of all genes, regardless of the HA concentration. We confirmed that HA decreased the expression of *E-cadherin* and increased the expression of *SNAIL* by day 21, but there was no change in the expression levels of pro-EMT genes (*SLUG, TWIST,* and *N-cadherin*) or other regulatory genes (*β-catenin* and *LEF1*). None of the expression levels correlated with HA concentration on days 7, 14, and 21.

Subsequently, we performed Western blot analysis and immunofluorescence staining for major EMT-related transcription factors (Snail1 and Twist) correlated with E-cadherin and N-cadherin on day 21 of undirected differentiation. The expression of E-cadherin, N-cadherin, Snail1, and Twist were compared in culture conditions without or with HA. In the control culture, the expression levels of E-cadherin were reduced, whereas those of N-cadherin and Twist were increased (Figures 2B, C). The aggregate and monolayer structures coexpressed E− and N-cadherins. High-magnification images show intense fluorescent labeling of EMT-related transcription factors such as Snail1 and Twist in the nuclei of cells with aggregated structures. However, in the culture with HA, the E-cadherin was expressed at high levels than in the control culture, but Twist was not detected. Snail1 and Twist were mainly localized in the cytoplasm, with a few cells exhibiting nuclear localization. No significant changes were observed in the expression of N-cadherin, Snail1, or Twist correlated with HA concentration. These results suggest that HA plays a crucial role in the suppression effect on the EMT which promotes differentiation from one phenotype to another during the undirected differentiation of iPSCs.

Previous studies have shown that BMP signaling plays a crucial role in promoting the ectoderm formation and TGF-β/Activin/Nodal signaling promotes the mesoendoderm formation (Gadue et al., 2006; Sumi et al., 2008; Payne et al., 2011; Lupo et al., 2013; Naujok et al., 2014). Activation of SMADs, major signal transducers in TGF-β and BMP signaling pathway has also been reported to be necessary for controlling the proliferation or differentiation of stem cells (Saha et al., 2008; Chambers et al., 2009). To determine whether HA suppressed the activation of the TGF-β/Activin/Nodal and BMP signaling during spontaneous differentiation, we assessed the expression of related genes by qRT-PCR. Compared to the undifferentiated iPSCs (day 0), the endogenous expression of *NODAL, TGFβ1*, *BMP4*, and *ACTIVINA* was significantly increased in control culture by day 21 (Figure 2D). In contrast, in cultures with HA, the cells maintained a lower expression level of all genes than in the control culture during the 21 days of culture. Moreover, it completely suppressed the expression of all genes, regardless of HA concentration. These results were then confirmed by immunofluorescence staining of phospho-SMAD1/5/8 (pSMAD1/5/8) and phospho-SMAD2/3 (pSMAD2/3) (Figure 2E). The cells in control culture showed both nuclear and cytoplasmic staining and the cells within the aggregates revealed nuclear positivity as well, demonstrating increased nuclear translocation of SMADs. However, the cells in cultures with HA showed cytoplasmic staining. The pSMAD2/3 protein were almost undetectable in both cultures. These results suggest that treatment with HA during the undirected differentiation of iPSCs suppressed the spatial heterogeneity of cells via the temporal distribution of E-cadherin-mediated cell-cell adhesion, resulting in the suppression of unwanted spontaneous differentiation of iPSCs through dual inhibition of TGF-β/Activin/Nodal signaling and BMP signaling (Figure 2F).

### Effect of HA on directed differentiation of iPSCs into neural progenitors in the presence of exogenous differentiation factors

**FIGURE 3:**
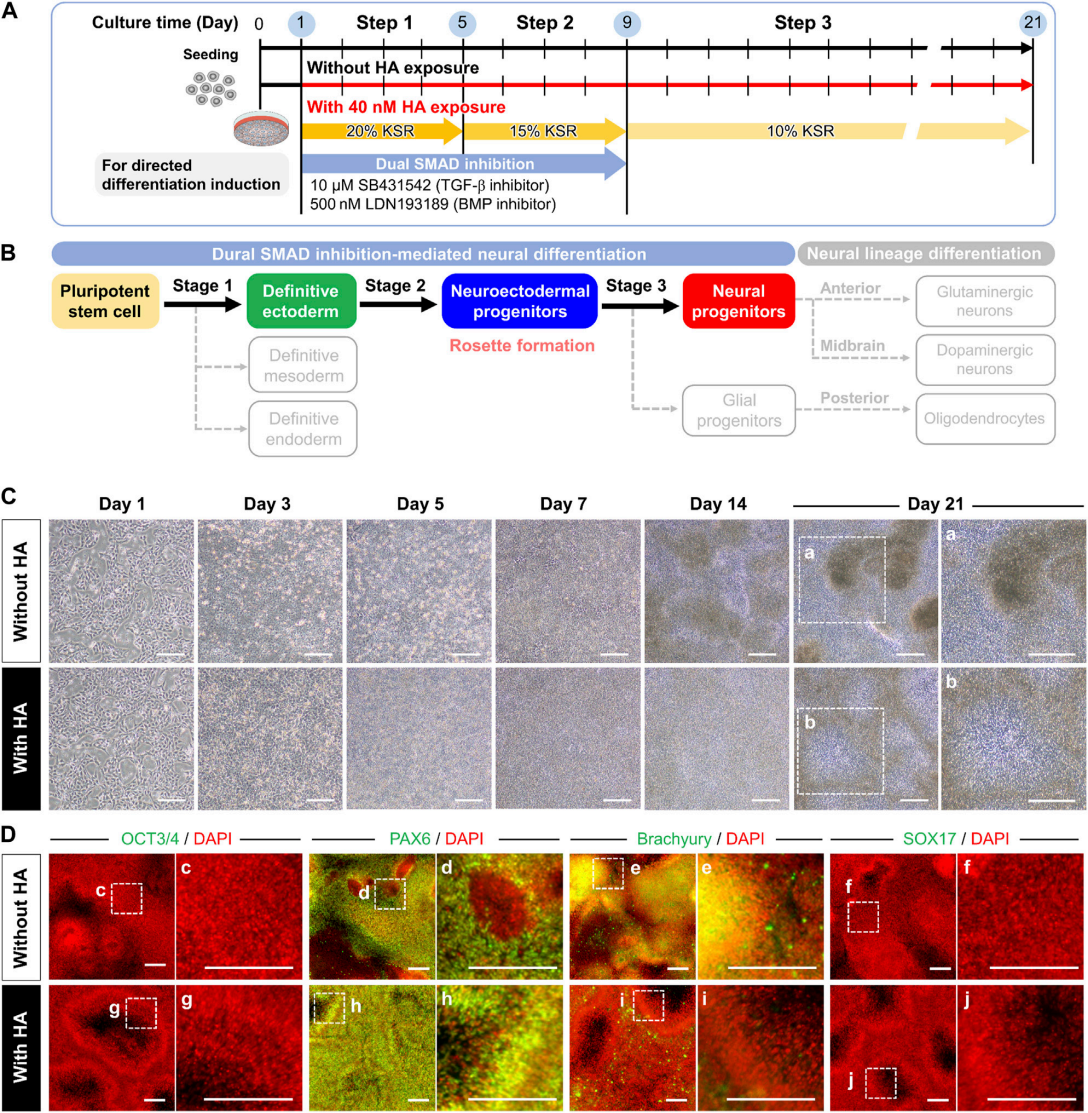
Morphological and phenotypic characterization of directed neural differentiation from iPSCs with or without HA. **(A)** Experimental scheme to examine the impact of HA exposure during directed neural progenitor differentiation from iPSCs. Cells were cultured on iMatrix-coated surface in a KSR-based medium containing LDN193189 (500 nM) and SB431542 (10 μM) from days 1–9. HA (40 nM) was added from days 1–21 of neural progenitor differentiation. **(B)** Schematic showing major steps for differentiating iPSCs into neural progenitors. Solid and dashed arrows indicate differentiation steps into target and non-target cells, respectively. **(C)** Phase-contrast image showing morphological characteristics in neural differentiation culture with and without HA exposure. Panels a and b are the enlarged images of the boxed area in the phase-contrast images. Scale bar, 1 mm. **(D)** Immunofluorescent images of a pluripotency marker (OCT3/4) and trilineage differentiation markers (PAX6, Brachyury, and SOX17) in neural progenitor differentiation without and with HA on day 21. Nuclei were stained with DAPI. Panels c–j are the enlarged images of the boxed area in the merged panels. Scale bars, 200 μm.

Because dual-SMAD inhibition is a well-established method for generating neural cells from iPSCs (Chambers et al., 2009), we investigated the effect of HA exposure on the dual SMAD inhibition of differentiation. iPSCs were initially cultured on iMartix-coated surfaces for 24 h and subsequently treated with KSR-based medium containing 500 nM LDN193189 and 10 μM SB431542 from days 1–9 (Figure 3A). Simultaneously, the cells were treated with or without 40 nM HA from days 1–21. Figure 3B shows the major steps involved in differentiating iPSCs into neural progenitors. In the untreated (control) culture, cells grew adherently, formed a monolayer of polygonal cells, and aggregated into clusters after they reached confluence on day 7, which grew heterogeneously (Figure 3C and Movie S1). From days 7–14, the confluent multi-layered culture and the level of spontaneous differentiation increased, indicating the appearance of a broad distribution within the culture. The rosettes began to form three-dimensional (3D) nerve islands that were maintained until day 21.

Treatment of iPSCs with HA caused drastic morphological changes in cells, with disruption of cell-cell contacts compared to the untreated (control) culture within 24 h, and cells exhibited cell growth and migration in a confluent state within 21 days (Figure 3C and Movie S1). Cells treated with HA had a greater ability to migrate than the untreated control cells, and their migration ability in the confluent state was almost the same over time. On day 7, the cells formed a confluent monolayer within 2 weeks of culture and multilayered structures with cell migration and division, in contrast to the monolayer structure of the cells when they reached excessive confluence. On day 14, the first signs of neural differentiation emerged as typical neuroepithelial structures or rosettes. On day 21, the cells exhibited a disk morphology of neural rosettes surrounding the central lumen.

**FIGURE 4:**
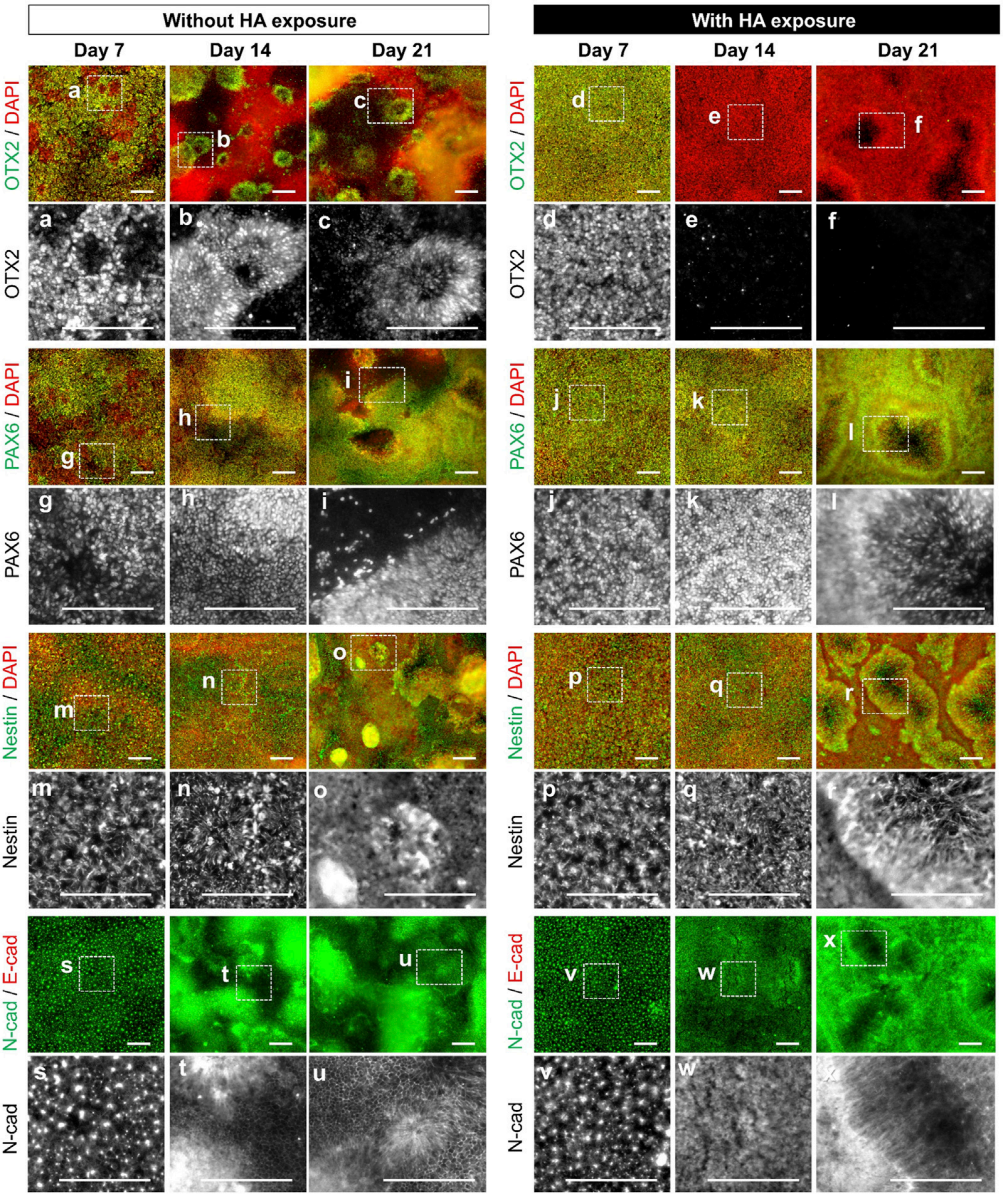
Characterization of directed neural differentiation from iPSCs with or without HA. Representative images of immunofluorescence staining for stage-specific markers (OTX2, PAX6, Nestin, and N-cadherin) at different time points (on days 7, 14, and 21) of neural progenitor differentiation without or with HA as shown in Figure 3A. Nuclei were stained with DAPI. Panels a–x are the enlarged images of the boxed area in the merged panels. Scale bars, 200 μm.

To confirm the neural differentiation of hiPSCs, we conducted time-course immunostaining using pluripotency-associated markers and early lineage specification-associated markers, including ectoderm, mesoderm, and endoderm, after 7, 14, and 21 days of differentiation. We observed a decrease in the pluripotency marker OCT3/4 on day 7 of differentiation under both culture conditions (Figure 3D). From days 7–14, all cultures acquired the neuroectoderm fate determinant PAX6, thereby diminishing the brachyury and SOX17 expression in the primitive striatal mesoderm and ectoderm. Furthermore, we confirmed the changes in the expression of the definitive ectodermal progenitor marker (OTX2) and neuroectodermal progenitor markers (PAX6, nestin, and N-cadherin) in cells cultured with and without HA (Figure 4). In the control culture, cells formed numerous aggregates ranging from a few cells to numerous cells during the 21 days of differentiation. Certain cells in the compact aggregate structures formed small rosettes expressing OTX2 and PAX6 on day 7 and their expression was maintained until day 21 of differentiation. In contrast, the cells cultured with HA developed directly into rosettes in confluent monolayers without aggregation. By day 7, cultures exposed to HA showed near-homogeneous monolayers of OTX2 and PAX6 expression, which produced cultures densely populated with neural rosettes. By day 14, the expression of OTX2 was almost completely absent in the monolayers, and PAX6 was strongly expressed. By day 21, most cells in the confluent monolayer organized in rosettes expressed PAX6. Furthermore, nestin and N-cadherin expression increased from days 7–14 of differentiation under both culture conditions. In the control culture, nestin was mainly located in the neural rosettes within the cell aggregates on day 21. High-magnification images showed that N-cadherin was found in cell aggregates or at sites of cell-cell contacts in confluent monolayers, and was elevated in cells in larger aggregates. In contrast, in cultures with HA, nestin was found in the neural rosettes, and N-cadherin was predominantly enriched in the inner circle of the rosette structures and was observed throughout the culture vessels. N-cadherin expression was mainly observed at the center of the rosettes on day 21.

**FIGURE 5:**
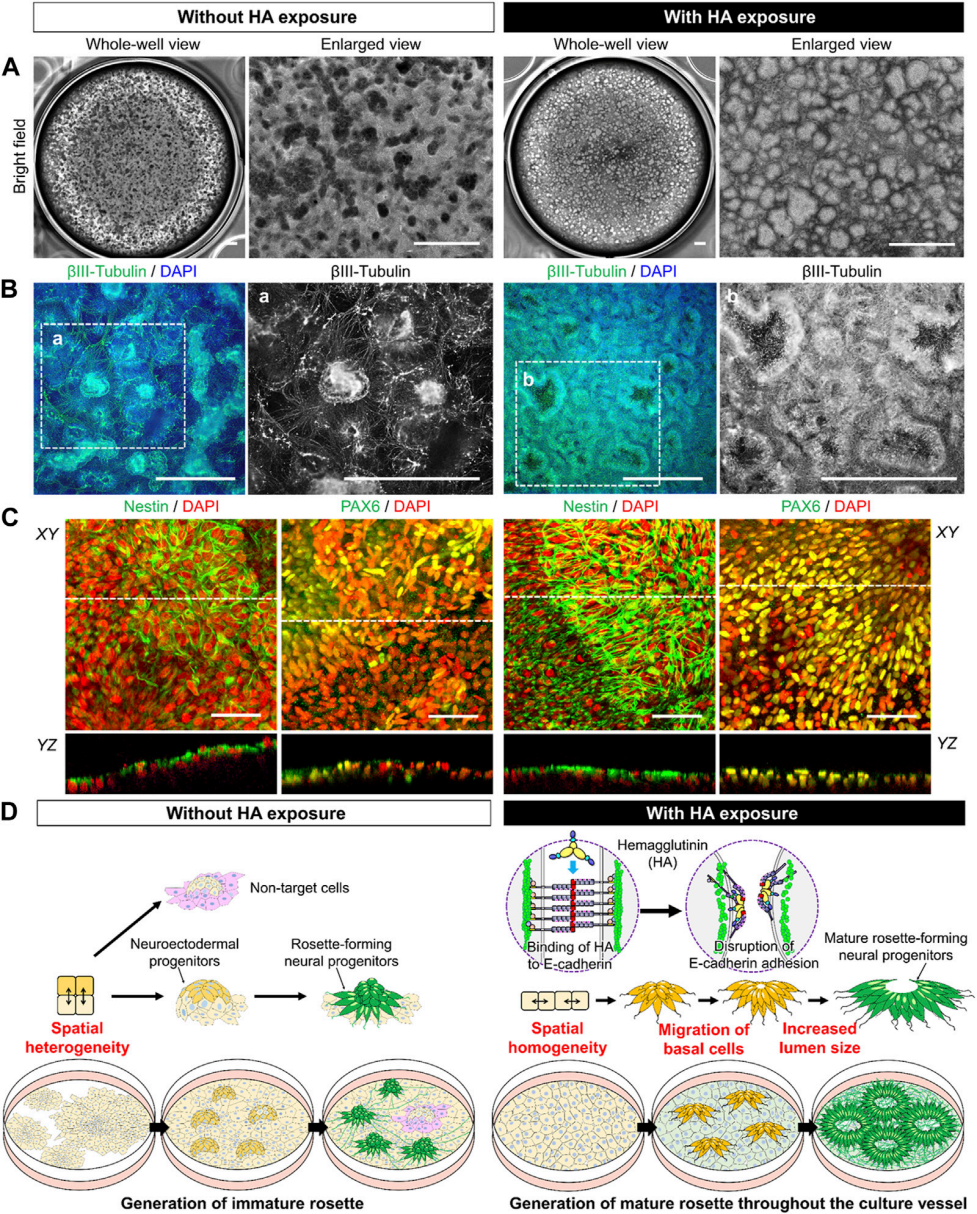
Characterization of iPSC-derived neural progenitor cell rosettes formed in cultures with or without HA. **(A)** Representative bright-field images showing morphological changes on day 21 of neural progenitor differentiation. Images are shown as the entire culture wells of a six-well plate and enlarged views. Scale bar, 1 mm. **(B)** Immunofluorescent images of neuronal marker, βIII-tubulin on day 21 of neural progenitor differentiation. Nuclei were stained with DAPI. Panels a–e are the enlarged images of the boxed area in the merged panels. Scale bars, 500 μm. **(C)** Immunofluorescent images of neuronal markers, Nestin and PAX6 on day 21 of neural progenitor differentiation. Nuclei were stained with DAPI. The image of confocal *YZ* plane through the dashed line in *XY* plane. Scale bar, 50 μm. **(D)** Schematic of the working hypothesis of induction in generation of functional iPSC-derived neural progenitor cell rosettes through HA-mediated E-cadherin disruption.

At the end of 21 days, we studied the expression of the neuron-specific marker βIII tubulin within a culture vessel and found that HA treatment produced morphologically distinct neural rosettes after 21 days of differentiation. Figure 5A shows the formation and enlarged views of neural rosettes throughout the six-well plate culture. In the control culture, the cells formed densely packed aggregates in the culture vessel, whereas in cultures with HA, the cells formed neural rosettes with large lumens distributed over the culture surface. Notably, we detected a difference in the neuron-specific marker βIII tubulin (Figure 5B). In the control culture, a few cells around the aggregates produced positive for βIII tubulin after induction of differentiation and most cells in the monolayer did not observe βIII tubulin. However, in cultures with HA, the βIII tubulin formed layers at the top and bottom of the lumen, and most cells were βIII tubulin positive neurons in the culture vessel. Further examination of the distribution of neural markers using confocal z-stack images and 3D reconstruction showed that nestin and PAX6 were mainly detected on the surface rosettes closest to the aggregated cells; however, certain cells within the aggregates did not express nestin or PAX6 (Figure 5C). In contrast, nestin and PAX6 were highly expressed within the neural rosette with the lumen in cultures containing HA. These results suggest that the use of HA for controlling spatial heterogeneity during neural differentiation involves the generation and migration of neural progenitors, resulting in the more effective formation of rosettes with a larger lumen throughout the culture vessel (Figure 5D).

**FIGURE 6:**
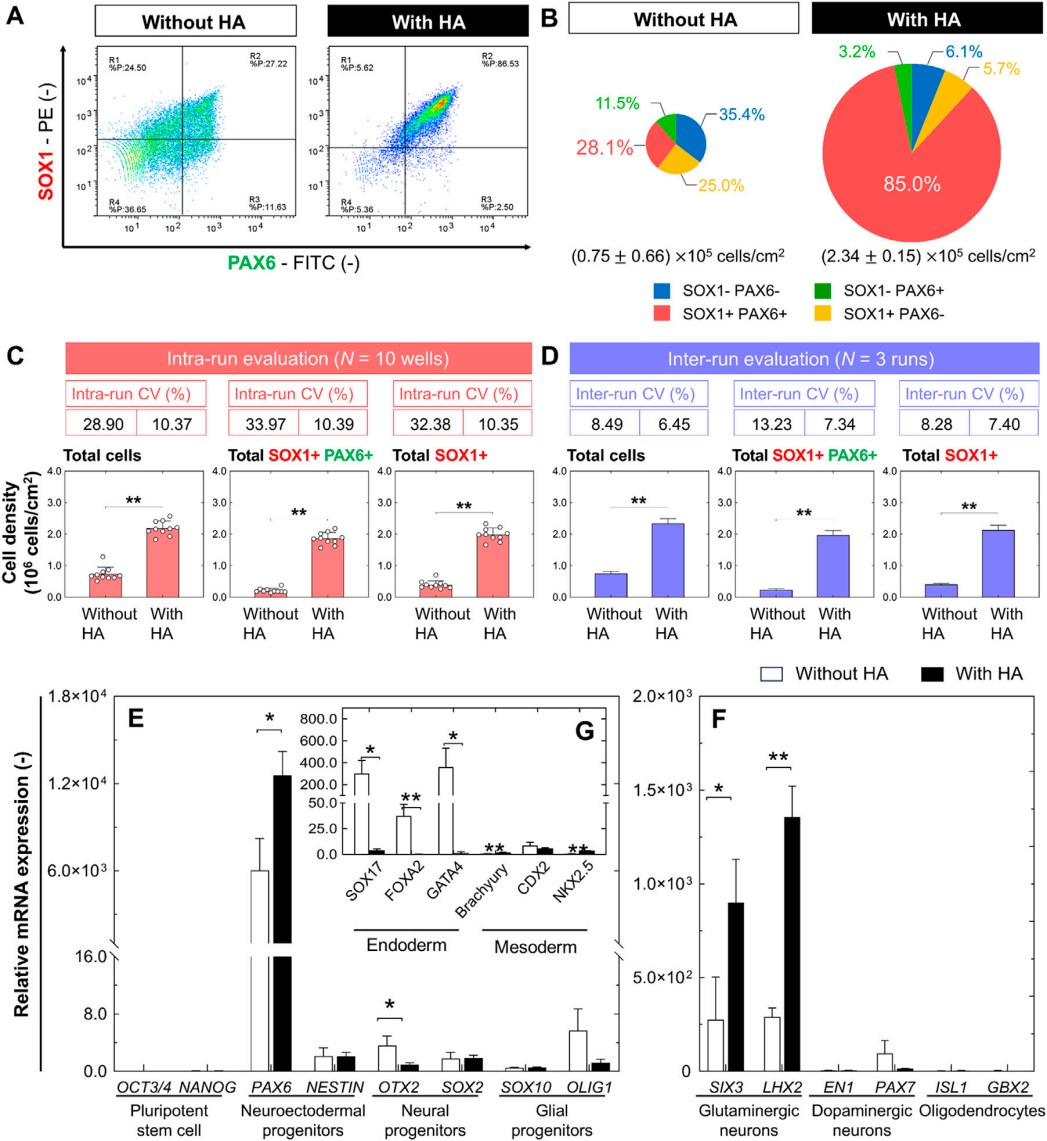
Comparison of efficiency and robustness of directed neural differentiation from iPSCs with or without HA. **(A)** Representative flow cytometry histograms and **(B)** pie charts of four different cell populations on day 21 of neural progenitor differentiation. Pie charts represent data obtained from ten culture wells in a single experiment. Size of pie charts represent total cell density. **(C,D)** Total cell density and densities of PAX6^+^/SOX1^+^ and PAX6^+^ cells, and intra-run and inter-run CV% on day 21 after neural progenitor differentiation. In-run CV% was calculated by culturing results from 10 culture wells as a single experimental run, and inter-run CV% was calculated by culturing results from 3 repeated runs of a single experimental run. Data are presented as mean ± SD. Comparisons were made using Student’s *t*-test (**p < 0.01, compared to the control). **(E,F)** Gene expression analysis by qRT-PCR for main pluripotency-associated genes (*OCT3/4* and *NANOG*), neuroectodermal progenitor-associated genes (*PAX6* and *NESTIN*), neural progenitor-associated genes (*OTX2* and *SOX2*), glial progenitor-associated genes (*SOX10* and *OLIG1*) as well as neural differentiation-associated genes of glutamatergic neuron (*SIX*3 and *LHX2*), dopaminergic neuron (*EN1* and *PAX7*), and oligodendrocyte (*ISL1* and *GBX2*). Data are presented as mean ± SD (*n* = 3). Significance was determined using Student’s *t*-test (**p < 0.01, *p < 0.05, compared to the control). **(G)** Gene expression analysis by qRT-PCR for early lineage specification-associated genes of endoderm (*SOX7, FOXA2,* and *GATA4*) and mesoderm (*Brachyury, CDX2,* and *NKX2.5*). Fold-expression for each mRNA obtained for each method was normalized to undifferentiated iPSCs on day 0. Data are presented as mean ± SD (*n* = 3). Significance was determined using Student’s *t*-test (**p < 0.01, *p < 0.05, compared to the control).

To evaluate the efficiency and robustness of neural differentiation, we performed flow cytometry analysis of the markers of neural progenitors, PAX6 and SOX1, after 21 days of differentiation. In the culture with HA, the total cell number was significantly higher than that in the control culture on day 21 (Figures 6A, B). The percentages of PAX6^+^ and PAX6^+^/SOX1^+^ cells showed that differentiation of iPSCs in culture with HA significantly increased the efficiencies of PAX6^+^/SOX1^+^ cells. The PAX6^+^ and PAX6^+^/SOX1^+^ cell percentages were 2.2 and 3.0-fold higher than those in the control culture, respectively. However, the PAX6^-^/SOX1^-^ cell percentage in control culture was significantly high, suggesting that spontaneous differentiation of iPSCs causes the formation of a heterogeneous cell population. The densities of PAX6^+^ and PAX6^+^/SOX1^+^cells at 21 days of differentiation were (1.93 ± 0.20)×10^5^ cells/cm^2^ and (1.86 ± 0.19)×10^5^ cells/cm^2^, indicating a 6.6-fold and 8.9-fold increase in cell density compared to control cultures, respectively (Figure 6C). Additionally, the intra- and inter-run CV% values obtained in cultures with HA were lower than those in the control, indicating that cultures with HA are stable and reliable across different cultures and runs. All inter-run CV% values were below 10%, indicating high consistency in terms of differentiation efficiency (Figure 6D). We further confirmed the maintenance of rosette structures with a central lumen formed in culture with HA after subsequent media change without HA, although the compact aggregate structures with small rosettes formed in the culture without HA could not be maintained in morphology (Supplementary Figure S1).

To determine neural differentiation efficiency, we quantified the expression levels of several pluripotency, mesodermal, endodermal, and neural progenitor genes using qRT-PCR after 21 days of differentiation. Compared to undifferentiated iPSCs (day 0), under both conditions, the expression of pluripotency-related genes (*OCT3/4* and *NANOG*) was drastically reduced by 21 days of differentiation, whereas the expression of neuroectodermal progenitor-related genes (*PAX6* and *NESTIN*), neural progenitor-associated genes (*OTX2* and *SOX2*), and glial progenitor-associated genes (*SOX10* and *OLIG1*) were increased (Figure 6E). Specifically, in cultures with HA, we observed a 2.1-fold increase of *PAX6*. Although there were no significant differences in the expression levels of dopaminergic neuron (*EN1* and *PAX7*) and oligodendrocyte (*ISL1* and *GBX2*) genes under both culture conditions, the expression of all genes was more variable than that in undifferentiated cells (Figure 6F). Notably, in cultures with HA, high expression of the glutamatergic neuron (*SIX*3 and *LHX2*) genes was specifically exhibited in iPSC-derived progenitors compared to control cultures, revealing the high capacity of neural progenitors to differentiate into mature glutamatergic neurons. We also confirmed that cells in culture with HA suppressed the expression of non-neural genes, including mesoderm (*Brachyury, CDX2,* and *NKX2.5*) and endoderm (*SOX7, FOXA2,* and *GATA4*) genes (Figure 6G), which was consistent with our immunofluorescence observations (Figure 2C).

## Discussion

The lack of efficient differentiation strategy for problem of PSC spontaneous differentiation into unwanted linages represents a serious obstacle to the clinical implementation of cell-based therapies. In this study, we developed a simple and robust method to overcome previously documented restrictions on the stability of iPSC differentiation into rosette-forming neural progenitors using HA. Addition of HA suppress the spatial heterogeneity of iPSCs through temporal disruption of E-cadherin-mediated cell-cell adhesion and subsequently leads to mechanical memory synchronization with YAP proteins (Kim et al., 2023). This, in turn increases the homogeneity of pluripotent cells. The underlying mechanism of cell synchronization during iPSC culture by HA has recently been explored; however, it is unclear whether the currently applied culture strategy allow robust neural differentiation from iPSCs. For a directed differentiation strategy involving the joint use of HA and dual SMAD inhibition, we hypothesized that i) HA-mediated temporal disruption of cell-cell interactions suppress spatial heterogeneity through regulation of cell behaviors within a culture by suppressing TGF-β/Activin/Nodal signaling and BMP signaling under undirected differentiation condition and ii) HA-mediated regulation of cell behaviors comprise the generation of neural progenitors under directed differentiation condition, resulting in the more effective formation of neural rosette with larger lumen throughout culture vessel. The main findings of this study are summarized below.

First, our findings demonstrated that HA treatment suppresses spontaneous differentiation of iPSCs in the absences of differentiation factors through inhibition of TGF-β/Activin/Nodal signaling and BMP signaling in cells. Temporal disruption of E-cadherin-mediated cell-cell adhesion by HA addition suppress the spatial heterogeneity of iPSCs through cell migration and subsequently leads to a morphologically homogeneous confluent monolayer without forming large aggregates (Figure 1). However, in the control culture, cells grew as a mixture of structures composed of larger aggregates and cell monolayer, showing a heterogeneous population of cells within the culture. The spatial heterogeneity of cells observed in the culture could be due to the cell behaviors that were dynamically changed, and that a mixture with confluent monolayers and multi-layered structures were established, which in turn induced different differentiation and proliferation of different cell lineages. The different localization of Rho-Rock-Myosin signaling in a PSC colony provides a positional cue that determines whether cells at a particular location within a culture should exit pluripotency and differentiate according to biochemical factors (Harb et al., 2008; Kim et al., 2022). In addition, changes in cell behavior associated with spatial locations frequently result in abnormal deformation that occurs at sites of nucleus-cytoskeletal linkage and cytoskeletal linkage to the ECM and adjacent cells (Dupont et al., 2011; Dasgupta and McCollum, 2019). Mechanical force generated by actin cytoskeleton enhances TGF-β1/Activin/Nodal and BMP activation and its effectors SMADs (Gadue et al., 2006; Lupo et al., 2013). This could eventually lead to new culture strategy of accelerating or manipulating the self-renewal and lineage-specific differentiation in PSCs (Gadue et al., 2006; Sumi et al., 2008; Payne et al., 2011; Lupo et al., 2013; Naujok et al., 2014; Zhang et al., 2018). These include the suppression of mesodermal and endodermal fates by inhibiting endogenous Activin and BMP signals and promoting neuralization of the primitive ectoderm by BMP inhibition. Consistent with previous observations, we found that the activation of TGF-β1/Activin/Nodal signaling and BMP signaling during spontaneous differentiation of iPSCs into unwanted lineages and suppression of spatial heterogeneity of cells by HA strikingly reduces spontaneous differentiation. Cells in culture with HA exhibited morphologically homogenous confluent monolayer with no signs of differentiation (Figure 1). These phenomena can be explained by observations that HA treatment leads to suppression of nuclear translocation of pSMAD1/5/8 (Figure 3). Therefore, when cell distribution was homogenized by the temporal disruption of E-cadherin-mediated cell-cell adhesion by added HA, TGF-β/Activin/Nodal signaling and BMP signaling linked SMAD signaling were suppressed and undesirable spontaneous differentiation of iPSCs was reduced.

Second, our findings demonstrated that HA treatment improves efficiency and reproducibility of existing differentiation method describing the generation of neural progenitors. The concept of Waddington’s epigenetic landscape of cell-fate determination has numerous branching valleys and ridges right from the start, with the process of cell fate determination (Kim and Kino-oka, 2018). When stem cells at the top of a mountain roll down like balls, obstacles guide the balls along specific paths. This provides insights into a possible reason for the uneven differentiation efficiency and poor reproducibility of current differentiation approaches. During the neural differentiation of PSCs, dual SMAD signaling inhibition enables the generation of a neural progenitor rosette-like structure with an apico-basal distribution of polar proteins similar to that described for neuroepithelial cells in the embryonic neural tube (Chambers et al., 2009; Lukovic et al., 2017). These structures comprise cells expressing the neural progenitor markers PAX6 and SOX1, and can differentiate into various region-specific neuronal and glial cell types in response to appropriate developmental cues. Application of HA to existing neural differentiation method using dual SMAD inhibition leads to the generation of generation of rosette-forming neural precursors with central lumen without the presence of other cells with pluripotent, mesodermal, or endodermal characteristics (Figures 4, 5). Cells cultured with HA form large networks of βIII tubulin + neurons throughout the culture vessels. We also confirmed higher percentages of PAX6^+^/SOX1^+^ and SOX1^+^ cells in cultures with HA (85.0% and 88.2%, respectively) than in control cultures (28.1% and 53.1%, respectively) (Figure 6). PAX6^+^/SOX1^+^ cells had a 5.1-fold higher yield of neural progenitor cells than the control cultures. The intra- and inter-run CV% values obtained in cultures with HA were lower than those in the control, indicating that HA can achieve relatively stable and reliable differentiation efficiency under directed differentiation conditions. They had greater expression of genes (*SIX*3 and *LHX2*) involved in developing glutamatergic neurons (Figure 6). These results indicate that controlling spatial heterogeneity by HA addition may not only stabilize the differentiation of PSC-derived neural progenitors, but also promote the differentiation yield and maturity of neural progenitors during directed differentiation of PSCs. Therefore, the combination of differentiation-stage-specific signal stimulation and HA is an efficient and stable strategy for inducing target cell populations and generating more functional cells.

Third, our findings demonstrated that HA treatment improves functional neural rosette formation with central lumen through control collective cell-cell behavior. During sequential neural differentiation in the ectoderm, upregulation of N-cadherin and loss of E-cadherin, known as cadherin switching, occur (Li et al., 2012). This highlights the possibility that the E-to N-cadherin switch during early neural induction regulates related signaling pathways that control cell behavior and influence differentiation. We observed two distinct types of rosettes in cultures with or without HA exposure (Figure 5). One type was a large rosette containing a central lumen that occurred stochastically in monolayer, and the other type was a small rosette lacking a central lumen that occurred in a dense aggregate. Dual SMAD inhibition incorporating HA considerably affected lumen formation in a neural rosette that exhibited N-cadherin expression (Figure 5). There appeared to be general features of N-cadherin that distinguished the culture with HA from the control culture. Although many strategies have allowed the generation of neural rosettes (Elkabetz et al., 2008; Harding et al., 2014; Malchenko et al., 2014; Ziv et al., 2015; Knight et al., 2018; Townshend et al., 2020), the potential for regional specification related to collective cell-cell behaviors has not been addressed. It has been demonstrated that control of collective cell-cell behaviors plays a pivotal role in the lumen formation within neural rosettes by regulating the actions of the adhesion complexes and cytoskeleton, including N-cadherin, F-actin, and myosin II (Elkabetz et al., 2008; Townshend et al., 2020). Previous studies used assistance of pharmacological agents and a quantitative live imaging to study the formation and maturation of rosette by PSC-derived neural progenitor cells, and demonstrated that basolaterally-driven cell movements precede the formation of apical rosette structures (Hříbková et al., 2018; Knight et al., 2018; Townshend et al., 2020). During these cell movements at basal side, central lumen emerges and expands, which was characterized by lumen formation in the apical region of the neural rosettes. The activation of the Notch pathway plays a key role in apical-basal cell polarity in maintenance of neural tube and ESC-derived neural rosette (Main et al., 2013). Consistent with previous observations, we found that under dual SMAD inhibition incorporating HA, lumen production for neural rosette formation was enhanced by basal cell migration during the loss of E-cadherin and expression of N-cadherin (Figure 5). There appeared to be general features of N-cadherin that distinguished the culture with HA from the control culture. N-cadherin was specifically concentrated in cells lining the lumens of forming rosettes, revealing that iPSC-derived neural progenitors can acquire a proper apicobasal organization despite being cultured in a 2D environment. Most of the neural rosettes with central lumen were maintained after subsequent media change without HA (Supplementary Figure S1). Collectively, our data reveal that HA combined with dual-SMAD inhibition is a highly efficient and robust strategy for generating functional iPSC-derived neural progenitor cell rosettes and HA can be used as a culture tool for neural differentiation and neural tube development.

## Conclusion

In this study, we developed a robust and efficient culture strategy for generating functional iPSC-derived neural progenitor cell rosettes, relying on a differentiation method that incorporates HA to control the spatial behavior of cells. Notably, we propose a simple and efficient way of enhancing the production of a large and homogeneous population of neural progenitor cells in directed neural differentiation, and demonstrate a culture strategy that uses HA to effectively generate functional neural rosettes with lumen throughout the culture vessel. Our findings may elucidate the impact of collective cell-cell behavior on rosette formation and maturation in a confluent culture. These findings may also help further improve the yield and quality of neurons for the generation of functional neural rosettes, providing a valid tool for neuronal disease modelling and drug discovery applications.

## Data Availability

The datasets presented in this study can be found in online repositories. The names of the repository/repositories and accession number(s) can be found in the article/Supplementary Material.
