# Supplementary material for: Stable and efficient generation of functional iPSC-derived neural progenitor cell rosettes through regulation of collective cell-cell behavior
Source: Front Bioeng Biotechnol. 2024 Jan 10;11:1269108. doi: 10.3389/fbioe.2023.1269108 (PMC10806250; doi:10.3389/fbioe.2023.1269108)
Supplement: Supplementary file 1 [file Table1.docx]

Supplementary Material

Supplementary Table 1: Antibodies used in the staining, western blotting, and flow cytometry experiments.

| **Antibodies or peptides** | **Source** | **Identifier** |
| --- | --- | --- |
| Mouse anti-OCT3/4 | Santa Cruz Biotechnology | sc-5279 |
| Rabbit anti-PAX6 | Sigma-Aldrich | SAB1410879 |
| Rabbit anti-SOX17 | Cell Signaling Technology | 81778s |
| Rabbit anti-Brachyury | Cell Signaling Technology | 81694s |
| Rabbit anti-E-cadherin | Cell Signaling Technology | 3195s |
| Mouse anti-N-cadherin | BD Biosciences | 610921 |
| Mouse anti-SNAIL1 | Santa Cruz Biotechnology | sc-271977 |
| Mouse anti-TWIST | Santa Cruz Biotechnology | sc-81417 |
| Mouse anti-OTX2 | Santa Cruz Biotechnology | sc-514195 |
| Rabbit anti-Nestin | Cell Signaling Technology | 73349 |
| Rabbit anti-βIII-Tubulin | Sigma-Aldrich | T2200 |
| Alexa Fluor 488-conjugated goat anti-mouse IgG antibody | ThermoFisher Scientific | A-11001 |
| Alexa Fluor 594-conjugated goat anti-mouse IgG antibody | ThermoFisher Scientific | A-11005 |
| Alexa Fluor 488-conjugated goat anti-rabbit IgG antibody | ThermoFisher Scientific | A-11008 |
| Alexa Fluor 594-conjugated goat anti-rabbit IgG antibody | ThermoFisher Scientific | A-11012 |
| DAPI | ThermoFisher Scientific | D3571 |
| Rabbit anti-phospho Smad1/Smad5/Smad8 (Ser463/465) | Merck | AB3848-I |
| Rabbit anti-phospho-SMAD2 (Ser465/467)/SMAD3 (Ser423/425) (D27F4) | Cell Signaling Technology | 8828S |
| StarBright Blue 700 Fluorescent-dye conjugated secondary antibody | BioRad | 12004158 |
| StarBright Blue 520 Fluorescent-dye conjugated secondary antibody | BioRad | 12005866 |
| Mouse anti-β-actin (8H10D10) | Cell Signaling Technology | 3700S |

Supplementary Table 2: Primers used in this study for qRT-PCR.

| **Genes** | **Primer sequences (5′–3′)** | **References** |
| --- | --- | --- |
| *E-Cadherin* | Forward: CCACCAAAGTCACGCTGAATAC  Reverse: GGAGTTGGGAAATGTGAGCAA | Perez et al., 2011 |
| *N-Cadherin* | Forward: CCACGCCGAGCCCCAGTATC  Reverse: CCCCCAGTCGTTCAGGTAATCA | Deng et al., 2017 |
| *β-Catenin* | Forward: AAAATGGCAGTGCGTTTAG  Reverse: TTTGAAGGCAGTCTGTCGTA | Gilbert-Sirieix et al., 2011 |
| *SNAIL1* | Forward: TTCCAGCAGCCCTACGACCAG  Reverse: GCCTTTCCCACTGTCCTCATC | Farfán et al., 2018 |
| *TWIST1* | Forward: GGAGTCCGCAGTCTTACGAG  Reverse: TCTGGAGGACCTGGTAGAGG | Kato et al., 2010 |
| *LEF1* | Forward: ACAGATCACCCCACCTCTTG  Reverse: TGAGGCTTCACGTGCATTAG | Wang et al., 2020 |
| *NODAL* | Forward: TGTTGGGGAGGAGTTTCATC  Reverse: GCACAACAAGTGGAAGGGAC | Vo and Khan, 2011 |
| *WNT3A* | Forward: CTTCTAATGGAGCCCCACCT  Reverse: GAGCCCAGAGATGTGTACTGC | Wang et al., 2017 |
| *BMP4* | Forward: TTCCTGGTAACCGAATGCTGA  Reverse: CCTGAATCTCGGCGACTTTTT | Liu et al., 2017 |
| *TGF-β* | Forward: CGCATCCTAGACCCTTTCTCCTC  Reverse: GGTGTCTCAGTATCCCACGGAAAT | Wan et al., 2018 |
| *OCT3/4* | Forward: CAAAACCCGGAGGAGGAGTC  Reverse: CACATCGGCCTGTGTATATC | Osakada et al., 2009 |
| *NANOG* | Forward: TGATTTGTGGGCCTGAAGAAAA  Reverse: GAGGCATCTCAGCAGAAGACA | Li et al., 2010 |
| *SOX2* | Forward: TGGCGAACCATCTCTGTGGT  Reverse: CCAACGGTGTCAACCTGCAT | Kim et al., 2009 |
| *SOX17* | Forward: GAGCCAAGGGCGAGTCCCGTA  Reverse: CCTTCCACGACTTGCCCAGCAT | Irie et al., 2015 |
| *FOXA2* | Forward: GTCCGACTGGAGCAGCTACTA  Reverse: GTACGTGTTCATGCCGTTCATC | Kuroda et al., 2019 |
| *GATA4* | Forward: TCCCAGACGTTCTCAGTCAG  Reverse: GGAGCTGGTCTGTGGAGACT | Mahmoud et al., 2016 |
| *BRACHYURY* | Forward: ACCTTCCATGTGAAGCAGCAA  Reverse: CTCCACAGTTGGGTTCATCTGTAA | Guo et al., 2018 |
| *CDX2* | Forward: TTCACTACAGTCGCTACATCACCAT  Reverse: TTGTTGATTTTCCTCTCCTTTGCT | Pinto et al., 2015 |
| *NKX2.5* | Forward: ACCCTGAGTCCCCTGGATTT  Reverse: TCACTCATTGCACGCTGCAT | Zwi et al., 2009 |
| *SOX10* | Forward: CCCGCACTACACCGACCA  Reverse: AGGAGAAAGCCGAGTAGA | Lai et al., 2021 |
| *OLIG1* | Forward: GCATGCAGGACCTGAACCT  Reverse: TATCTTGGAGAGCTTGCGGC | García-León et al., 2018 |
| *SIX3* | Forward: CCTCCCACTTCTTGTTGCCA  Reverse: CGCTACTCGCCAGAAGTATGG | Aijaz et al., 2005 |
| *LHX2* | Forward: CCAAGGACTTGAAGCAGCTC  Reverse: AAGAGGTTGCGCCTGAACT | Rhee et al., 2019 |
| *EN1* | Forward: GGACAATGACGTTGAAACGCAGCA  Reverse: AAGGTCGTAAGCGGTTTGGCTAGA | Rhee et al., 2019 |
| *PAX7* | Forward: ACCCCTGCCTAACCACATC  Reverse: GCGGCAAAGAATCTTGGAGAC | Al Tanoury et al., 2020 |
| *ISL1* | Forward: AGCAGCCCAATGACAAAACT  Reverse: CTGAAAAATTGACCAGTTGCTG | Rhee et al., 2019 |
| *GBX2* | Forward: CACCACGTCTACGGGCAAGAAC  Reverse: AGCTGCTGATGCTGACTTCTGA | Rhee et al., 2019 |
| *β-ACTIN* | Forward: CATGTACGTTGCTATCCAGGC  Reverse: CTCCTTAATGTCACGCACGAT | Wang et al., 2017 |

**Supplemental References**

Aijaz, S., Allen, J., Tregidgo, R., van Heyningen, V., Hanson, I., Clark, B.J. (2005). Expression analysis of SIX3 and SIX6 in human tissues reveals differences in expression and a novel correlation between the expression of SIX3 and the genes encoding isocitrate dehyhrogenase and cadherin 18. Genomics *86,* 86–99. https://doi: 10.1016/j.ygeno.2005.03.002.

Al Tanoury, Z., Rao, J., Tassy, O., Gobert, B., Gapon, S., Garnier, J.M., Wagner, E., Hick, A., Hall, A., Gussoni, E., Pourquié, O. (2020). Differentiation of the human PAX7-positive myogenic precursors/satellite cell lineage in vitro. Development *147,* dev187344. https://doi: 10.1242/dev.187344.

Deng, G., Zeng, S., Ma, J., Zhang, Y., Qu, Y., Han, Y., Yin, L., Cai, C., Guo, C., Shen, H. (2017). The anti-tumor activities of Neferine on cell invasion and oxaliplatin sensitivity regulated by EMT via Snail signaling in hepatocellular carcinoma. Sci. Rep. *7*, 41616. https://doi.org/10.1038/srep41616.

Farfán, N., Ocarez, N., Castellón, E. A., Mejía, N., de Herreros, A. G., Contreras, H. R. (2018). The transcriptional factor ZEB1 represses Syndecan 1 expression in prostate cancer. Sci. Rep. *8,* 11467. https://doi.org/10.1038/s41598-018-29829-1.

García-León, J.A., Kumar, M., Boon, R., Chau, D., One, J., Wolfs, E., Eggermont, K., Berckmans, P., Gunhanlar, N., de Vrij, F., Lendemeijer, B., Pavie, B., Corthout, N., Kushner, S.A., Dávila, J.C., Lambrichts, I., Hu, W.S., Verfaillie, C.M. (2018). SOX10 single transcription factor-based fast and efficient generation of oligodendrocytes from human pluripotent stem cells. Stem Cell Reports *10*, 655–672. https://doi: 10.1016/j.stemcr.2017.12.014.

Gilbert-Sirieix M., Makoukji J., Kimura S., Talbot M., Caillou B., Massaad C., Massaad-Massade L. (2011). Wnt/β-catenin signaling pathway is a direct enhancer of thyroid transcription factor-1 in human papillary thyroid carcinoma cells. PLoS One *6*, e22280. https://doi.org/10.1371/journal.pone.0022280.

Guo, Y., Zeng, Q., Liu, S., Yu, Q., Wang, P., Ma, H., Shi, S., Yan, X., Cui, Z., Xie, M., Xue, Y., Zha, Q., Li, Z., Zhang, J., Tang, S., Chen, J. (2018). Generation of an iPS cell line via a non-integrative method using urine-derived cells from a patient with USH2A-associated retinitis pigmentosa. Stem Cell Res. *29,* 139–142. https://doi.org/10.1016/j.scr.2018.03.022

Irie, N., Weinberger, L., Tang, W.W.C., Kobayashi, T., Viukov, S., Manor, Y.S., Dietmann, S., Hanna, J.H., Surani, M.A. (2015). SOX17 is a critical specifier of human primordial germ cell fate. Cell *160,* 253–268. https://doi.org/10.1016/j.cell.2014.12.013

Kim, J.B., Greber, B., Arazo-Bravo, M.J., Meyer, J., Park, K.I., Zaehres, H., Schöler, H.R. (2009). Direct reprogramming of human neural stem cells by OCT4. Nature *214,* 102284. https://doi.org/10.1038/nature08436.

Kato, Y., Yashiro, M., Noda, S., Tendo, M., Kashiwagi, S., Doi, Y., Nishii, T., Matsuoka, J., Fuyuhiro, Y., Shinto, O., Sawada, T., Ohira, M., Hirakawa, K. (2010). Establishment and characterization of a new hypoxia-resistant cancer cell line, OCUM-12/Hypo, derived from a scirrhous gastric carcinoma. Br. J. Cancer *102,* 898–907. https://doi.org/10.1038/sj.bjc.6605543.

Kuroda, T., Yasuda, S., Tachi, S., Matsuyama, S., Kusakawa, S., Tano, K., Miura, T., Matsuyama, A., Sato, Y. (2019). SALL3 expression balance underlies lineage biases in human induced pluripotent stem cell differentiation. Nat. Commun. *10,* 2175. https://doi.org/10.1038/s41467-019-09511-4

Lai, X., Liu, J., Zou, Z., Wang, Y., Wang, Y., Liu, X., Huang, W., Ma, Y., Chen, Q., Li, F., Wu, G., L,i W., Wang, W., Yuan, Y., Jiang, B. (2021). SOX10 ablation severely impairs the generation of postmigratory neural crest from human pluripotent stem cells. Cell Death Dis. *12,* 814. https://doi: 10.1038/s41419-021-04099-4.

Li, D., Zhou, J., Wang, L., Shin, M.E., Su, P., Lei, X., Kuang, H., Guo, W., Yang, H., Cheng, L., Tanaka, T.S., Leckband, D.E., Reynolds, A.B., Duan, E., Wang, F. (2010). Integrated biochemical and mechanical signals regulate multifaceted human embryonic stem cell functions. J. Cell Biol. *191,* 631–644. https://doi.org/10.1083/jcb.201006094.

Liu, Y., Du, S.Y., Ding, M., Dou, X., Zhang, F.F., Wu, Z.Y., Qian, S.W., Zhang, W., Tang, Q.Q., Xu, C.J. (2017). The BMP4-Smad signaling pathway regulates hyperandrogenism development in a female mouse model. J. Biol. Chem. *292,* 11740–11750. https://doi: 10.1074/jbc.M117.781369.

Mahmoud, M. M., Kim, H. R., Xing, R., Hsiao, S., Mammoto, A., Chen, J., Serbanovic-Canic, J., Feng, S., Bowden, N. P., Maguire, R., Ariaans, M., Francis, S. E., Weinberg, P. D., van der Heiden, K., Jones, E. A., Chico, T. J., Ridger, V., Evans, P. C. (2016). TWIST1 integrates endothelial responses to flow in vascular dysfunction and atherosclerosis. Circ. Res. *119,* 450–462. <https://doi.org/10.1161/circresaha.116.308870>

Osakada, F., Jin, Z.B., Hirami, Y., Ikeda, H., Danjyo, T., Watanabe, K., Sasai, Y., & Takahashi, M. (2009). In vitro differentiation of retinal cells from human pluripotent stem cells by small-molecule induction. J. Cell Sci. *122,* 3169–3179. https://doi.org/10.1242/jcs.050393.

Perez, R. E., Navarro, A., Rezaiekhaligh, M. H., Mabry, S. M., Ekekezie, I. I. (2011). TRIP-1 regulates TGF-β1-induced epithelial-mesenchymal transition of human lung epithelial cell line A549. Am. J. Physiol. Lung Cell Mol. Physiol. *300*, L799–807. https://doi.org/10.1152/ajplung.00350.2010.

Pinto, R., Barros, R., Pereira-Castro, I., Mesquita, P., Da Costa, L.T., Bennett, E.P., Almeida, R., David, L. (2015). CDX2 homeoprotein is involved in the regulation of ST6GalNAc-I gene in intestinal metaplasia. Lab. Investig. *95,* 718–727. https://doi.org/10.1038/labinvest.2015.52

Rhee, Y.H., Puspita, L., Sulistio, Y.A., Kim, S.W., Vidyawan, V., Elvira, R., Chang, M.Y., Shim, J.W., Lee, S.H. (2019). Efficient neural differentiation of hPSCs by extrinsic signals derived from co-cultured neural stem or precursor cells. Mol. Ther. *27,* 1299–1312. https://doi: 10.1016/j.ymthe.2019.04.011.

Vo, B.T., Khan, S.A. (2011) Expression of nodal and nodal receptors in prostate stem cells and prostate cancer cells: autocrine effects on cell proliferation and migration. Prostate *71*, 1084–1096. https://doi: 10.1002/pros.21326.

Wan, S., Fu, X., Ji, Y., Li, M., Shi, X., Wang, Y. (2018). FAK- and YAP/TAZ dependent mechanotransduction pathways are required for enhanced immunomodulatory properties of adipose-derived mesenchymal stem cells induced by aligned fibrous scaffolds. Biomaterials *171,* 107–117. https://doi: 10.1016/j.biomaterials.2018.04.035.

Wang, H., Bender, A., Wang, P., Karakose, E., Inabnet, W.B., Libutti, S.K., Arnold, A., Lambertini, L., Stang, M., Chen, H., Kasai, Y., Mahajan, M., Kinoshita, Y., Fernandez-Ranvier, G., Becker, T.C., Takane, K.K., Walker, L.A., Saul, S., Chen, R., Scott, D.K., Ferrer, J., Antipin, Y., Donovan, M., Uzilov, A. V., Reva, B., Schadt, E.E., Losic, B., Argmann, C., Stewart, A.F. (2017). Insights into beta cell regeneration for diabetes via integration of molecular landscapes in human insulinomas. Nat. Commun. *8,* 767. <https://doi.org/10.1038/s41467-017-00992-9>.

Wang, T., Hao, Z., Liu, C., Yuan, L., Li, L., Yin, M., Li, Q., Qi, Z., Wang, Z. (2020). LEF1 mediates osteoarthritis progression through circRNF121/miR-665/MYD88 axis via NF-кB signaling pathway. Cell Death Dis. *11,* 598. <https://doi.org/10.1038/s41419-020-02769-3>.

Wang, Q., Zou, Y., Nowotschin, S., Kim, S.Y., Li, Q.V., Soh, C.L., Su, J., Zhang, C., Shu, W., Xi, Q., Huangfu, D., Hadjantonakis, A.K., Massagué, J. (2017). The p53 family coordinates Wnt and Nodal inputs in mesendodermal differentiation of embryonic stem cells. Cell Stem Cell *20,* 70–86. https://doi: 10.1016/j.stem.2016.10.002.

Zwi, L., Caspi, O., Arbel, G., Huber, I., Gepstein, A., Park, I.H., Gepstein, L. (2009). Cardiomyocyte differentiation of human induced pluripotent stem cells. Circulation *120,* 1513–1523. https://doi: 10.1161/CIRCULATIONAHA.109.868885.
