# Supplementary material for: Stable and efficient generation of functional iPSC-derived neural progenitor cell rosettes through regulation of collective cell-cell behavior
Source: Front Bioeng Biotechnol. 2024 Jan 10;11:1269108. doi: 10.3389/fbioe.2023.1269108 (PMC10806250; doi:10.3389/fbioe.2023.1269108)
Supplement: Supplementary file 3 [file DataSheet1.docx]

Supplementary Material


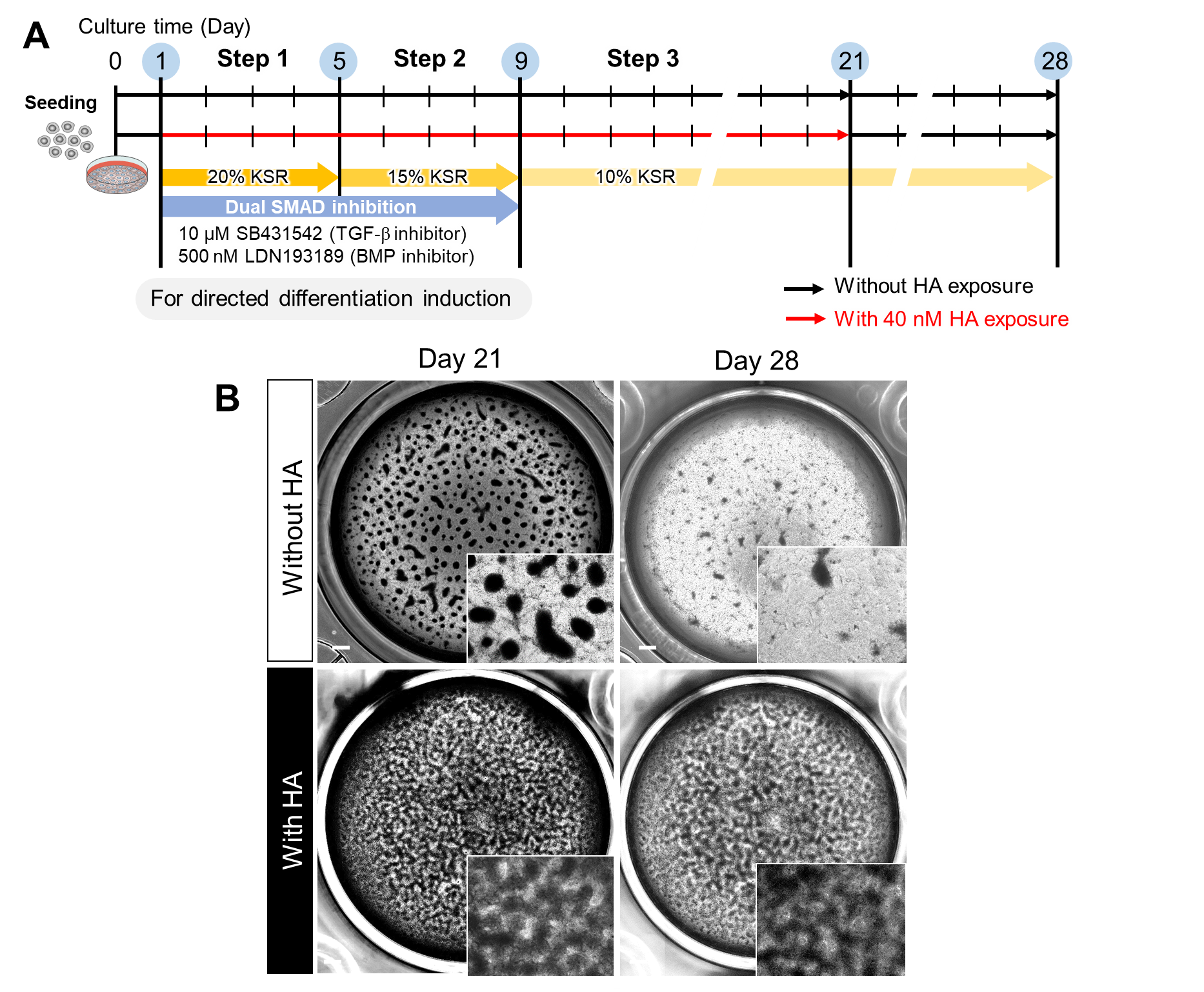


**Supplementary Figure 1. Comparison of stabilization of rosette structures formed in cultures with or without HA after subsequent media change without HA.** (A) Experimental scheme to examine the stabilization of rosette structures formed in culture with or without HA exposure. Cells were cultured on iMatrix-coated surface in a KSR-based medium containing LDN193189 (500 nM) and SB431542 (10 μM) from days 1 to 9. HA (40 nM) was added to the medium from day 1 to day 21 of differentiation, and HA was not added from day 21 to day 28 of culture. (B) Whole-well image from 6-well plate and enlarged images in phase-contrast image showing stabilization of rosette structures formed in cultures with or without HA after subsequent media change without HA. Scale bar, 1 mm.
